# Where and When Bacterial Chromosome Replication Starts: A Single Cell Perspective

**DOI:** 10.3389/fmicb.2018.02819

**Published:** 2018-11-26

**Authors:** Damian Trojanowski, Joanna Hołówka, Jolanta Zakrzewska-Czerwińska

**Affiliations:** Department of Molecular Microbiology, Faculty of Biotechnology, University of Wrocław, Wrocław, Poland

**Keywords:** replication initiation, *oriC*, replisome, single-cell, bacterial chromosome

## Abstract

Bacterial chromosomes have a single, unique replication origin (named *oriC*), from which DNA synthesis starts. This study describes methods of visualizing *oriC* regions and the chromosome replication in single living bacterial cells in real-time. This review also discusses the impact of live cell imaging techniques on understanding of chromosome replication dynamics, particularly at the initiation step, in different species of bacteria.

## Introduction

DNA replication is an enormously intricate process, in which a few dozen enzymes catalyze a series of reactions, including DNA unwinding and the synthesis of sister DNA strands. This process must be highly precise and accurately timed to prevent any unnecessary loss of energy and to ensure that DNA is faithfully and completely replicated only once per cell-division cycle (Leonard and Grimwade, 2015). In all three domains of life, chromosomal replication is mainly regulated at the initiation step (Nielsen and Løbner-Olesen, 2008; Aves, 2009; Skarstad and Katayama, 2013), an important cell cycle checkpoint guaranteeing that DNA replication begins at the right place and time.

**FIGURE 1:**
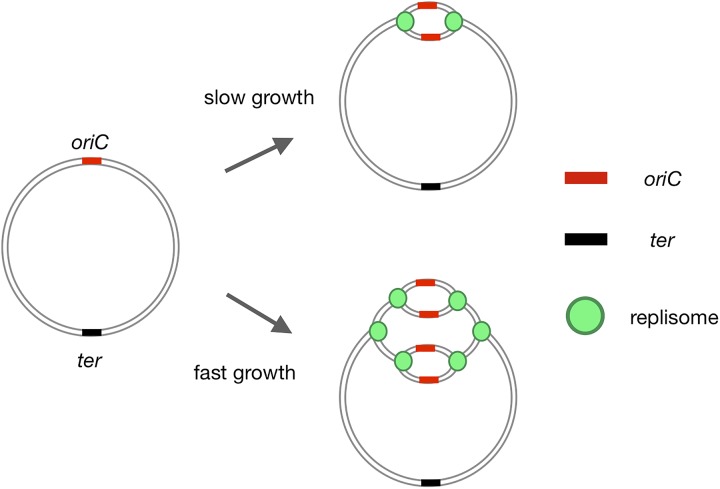
Initiation of bacterial replication. Replication of the bacterial chromosome is initiated at a single *oriC* region, proceeds in both directions, and terminates at the *ter* region. During slow growth, replication is initiated once per cell cycle. In fast growers under optimal conditions, another round of replication is initiated before the previous round has been completed, resulting in the inheritance by daughter cells of partially replicated chromosomes.

Most bacterial genomes consist of one covalently closed chromosome (Figure 1). In a few bacteria, however, the genetic information is distributed on two [e.g., *Vibrio cholerae* (Trucksis et al., 1998)] or even more [e.g., *Paracoccus denitrificans* (Winterstein and Ludwig, 1998)] chromosomes. Interestingly, some bacteria possess linear chromosomes [e.g., *Streptomyces* (Lin et al., 1993)].

**FIGURE 2:**
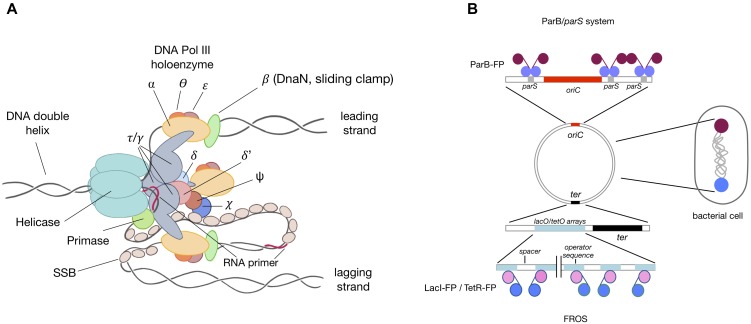
Replisome structure and localization. **(A)** Schematic diagram of a replisome. A replisome is a multiprotein complex involved in DNA replication. A helicase unwinds the chromosome, separating the two single-stranded DNA strands. The leading strand is synthesized continuously, while the lagging strand is synthesized in approximately 1 kbp fragments, starting from the short primers added by the primase. The three core polymerases are loaded into each replication fork by the clamp loader and bind to the sliding clamp, enabling high activity of the entire replisome. **(B)** Schematic localization of chromosomal loci using ParB/*parS* and FROS system. Under optimal conditions (a bacterial cell with a longitudinal chromosome conformation), ParB-FP binds to *parS* sequences (purple) in the *oriC* region, while the *ter* region (blue) is labeled through insertion of operator arrays and subsequent binding of repressor-FP.

In contrast to eukaryotes, bacterial chromosomes have a single, unique origin of replication (*oriC*) (Bird et al., 1972; Kaguni and Kornberg, 1984; Gao and Zhang, 2008; Masai et al., 2010; Méchali, 2010; Katayama, 2017). DNA synthesis is initiated at this unique *oriC*, generating a single replication eye per chromosome (Figure 1). Cooperative binding of the initiator protein, DnaA, to multiple DnaA-recognition sites (DnaA boxes) within the *oriC* region triggers separation of the DNA strands at the DNA unwinding element (DUE), providing an entry site for the machinery of replication (replisome, Figures 1, 2A; Skarstad et al., 1986, 1990; Bach et al., 2008; Leonard and Grimwade, 2011; Wolański et al., 2014; Richardson et al., 2016).

Enormous progress has been made in recent years toward understanding the mechanisms of replication initiation, particularly the organization and function of *oriC* regions in different bacteria (Donczew et al., 2012; Makowski et al., 2016; Jaworski et al., 2018; Midgley-Smith et al., 2018; Samadpour and Merrikh, 2018). Less is known, however, about the subcellular localization of replication processes during the cell cycle in various bacterial species. The development of sophisticated cell biology techniques has allowed examination of when and where the replication machinery is assembled within the bacterial cells, and how the initiation of replication is coordinated with the cell cycle (Donczew et al., 2012; Harms et al., 2013; Santi and McKinney, 2015; Trojanowski et al., 2015; Böhm et al., 2017). This process is particularly interesting in bacteria with two chromosomes (*V. cholerae*) (Demarre et al., 2014; Ramachandran et al., 2018) and in those that undergo complex cell differentiation (*Caulobacter crescentus*) (Jensen et al., 2001; Toro et al., 2008) and/or exhibit complicated life cycles, e.g., *Myxococcus xanthus* (Harms et al., 2013; Lin et al., 2017) and *Streptomyces* species (Kois-Ostrowska et al., 2016). In these bacteria, the regulatory networks that control replication initiation are likely to be intricate and require specific mechanisms that can synchronize the initiation of chromosomal replication with developmental processes.

The main goal of this review is to highlight imaging techniques that allow the determination of the subcellular location of *oriC* regions and the initiation of chromosome replication (i.e., assembly of the replication machinery) in single living bacterial cells in real time. This review also discusses the impact of real-time single-cell imaging on understanding of chromosome replication dynamics, particularly at the initiation step, in different bacteria.

## Visualization of Replication Initiation and Replisome Dynamics in Live Cells

The development of live cell imaging techniques has allowed the visualization of replisomes (Figure 2A; Jensen et al., 2001; Reyes-Lamothe et al., 2008; Wang and Sherratt, 2010; Harms et al., 2013; Santi and McKinney, 2015; Trojanowski et al., 2015; Mangiameli et al., 2017) in live cells and the study of DNA replication dynamics, including the timing and localization of replication initiation, in real time at the single-cell level. Microscopic analysis of live cells has several advantages over analysis of fixed samples. Fixing the cells, a process that involves dehydration and/or intracellular cross-linking, may influence the localization of proteins or subcellular structures of interest. Moreover, some fusions with fluorescent proteins (FP) are sensitive to the harsh conditions used during fixation. For example, different sample preparation of *Mycobacterium smegmatis* cells results in ParA-EGFP localizing either apically or as a cloud arising from the new cell pole (Ginda et al., 2013, 2017). Furthermore, permeabilization of the bacterial cell wall during immunostaining may contribute to a loss of cytoplasmic content or, due to cellular crowding, may generate high background noise or alter the localization of large immunocomplexes, particularly when using secondary antibodies for signal amplification. Although several high quality studies of fixed samples have provided invaluable data, the conditions found in cells fixed on a coverslip only approximate the conditions found in live cells.

Replication is visualized primarily by the fusion of different replisome (DNA polymerase III) subunits (Figure 2A) to a variety of FP. The choice of subunit to create the fusion protein should be guided by the specific application and the specific type of bacterium. *Escherichia coli* is the best characterized bacterial model for tracking live replication (Kongsuwan et al., 2002; Bates and Kleckner, 2005; Fossum et al., 2007; Reyes-Lamothe et al., 2008, 2010; Su’etsugu and Errington, 2011; Wang et al., 2011; Moolman et al., 2014; Beattie et al., 2017). However, several reports have tracked replication in other organisms, including *Bacillus subtilis* (Lemon and Grossman, 1998; Migocki et al., 2004; Berkmen and Grossman, 2006; Mangiameli et al., 2017; Li et al., 2018), *C. crescentus* (Jensen et al., 2001; Fernandez-Fernandez et al., 2013; Arias-Cartin et al., 2017), *V. cholerae* (Srivastava and Chattoraj, 2007; Stokke et al., 2011), *M. smegmatis* (Santi et al., 2013; Santi and McKinney, 2015; Trojanowski et al., 2015, 2017), *Streptomyces coelicolor* (Ruban-Ośmiałowska et al., 2006; Wolański et al., 2011), *Corynebacterium glutamicum* (Böhm et al., 2017), *Pseudomonas aeruginosa* (Vallet-Gely and Boccard, 2013), *M. xanthus* (Harms et al., 2013), and *Streptococcus pneumoniae* (Raaphorst et al., 2017). Findings of these studies may help in the construction of fluorescent fusions of replisome components in other bacteria. It is also important to consider alternative N- and C-terminal fusion, as one, or sometimes both, ends of target proteins may be implicated in inter- or intra-molecular interactions. The sliding clamp (Figure 2A) is the protein of choice in most studies and both N- and C-terminal fusions proved to be functional in a range of species (Kongsuwan et al., 2002; Reyes-Lamothe et al., 2010; Su’etsugu and Errington, 2011; Moolman et al., 2014; Santi and McKinney, 2015; Trojanowski et al., 2015; Arias-Cartin et al., 2017; Böhm et al., 2017; Mangiameli et al., 2017; Hołówka et al., 2018). However, the sliding clamp also participates in processes other than DNA replication, including recombination and DNA repair, possibly altering the distribution of DnaN-FP (or FP-DnaN) foci in these cells. This is not usually a concern in wild-type-like fluorescent reporter strains, under both optimal and minimal conditions, but may be of concern in knock-out/overproducing mutant strains, involving, for example, genes engaged in DNA repair, or when studying replication dynamics under stress-inducing conditions such as in the presence of antibiotics, mutagenic compounds like mitomycin, and replication inhibitors. In these experiments, choosing another replisome component may be advisable. Beside the siding clamp, DnaX (Lemon and Grossman, 2000; Bates and Kleckner, 2005; Berkmen and Grossman, 2006; Vallet-Gely and Boccard, 2013; Raaphorst et al., 2017) (particularly its C-terminal fusion) is frequently used as a replisome localization marker. The *dnaX* gene encodes two alternative proteins, τ – the full-length protein encoded by the *dnaX* gene, and γ, which originates from ribosome switching during translation, resulting in premature termination of translation and generating a truncated protein. Single-stranded DNA binding protein (SSB) (Figure 2A) has also been tested in several studies (Reyes-Lamothe et al., 2008, 2010; Harms et al., 2013; Sukumar et al., 2014; Santi and McKinney, 2015; Mangiameli et al., 2017; Raaphorst et al., 2017). Monitoring replisome dynamics in strains expressing fusion proteins encoded on an episomal plasmid is not recommended, as plasmid replication is triggered mainly by the same protein components that trigger chromosomal replication. Fusion with catalytic core subunits (Lemon and Grossman, 1998; Migocki et al., 2004; Trojanowski et al., 2017) is also possible, although additional cargo attached to core Pol-DNA III may affect nucleotide incorporation rates and influence the kinetic parameters of the entire replication complex. This was shown for *M. smegmatis*, where the C-terminal fusion of a catalytic alpha subunit to EYFP prolonged the C-period (Trojanowski et al., 2017). Thus proteins other than the catalytic core complex may be a better choice for studies of replisome dynamics. Other fusions successfully used for replisome tracking include DnaB (DNA helicase) (Jensen et al., 2001; Beattie et al., 2017), DnaQ (Reyes-Lamothe et al., 2008, 2010; Wallden et al., 2016; Mangiameli et al., 2017), and χ and δ′ subunits (Jensen et al., 2001; Reyes-Lamothe et al., 2008). When designing a fluorescent fusion for replisome visualization, additional features should be taken into account, especially oligomerization status, fluorescence yield and spectral properties. FP (especially GFP derivatives) are likely to form low-affinity oligomers (Costantini et al., 2012), which may influence the dynamics of the studied protein complex, especially when the fusion protein is produced at a high level. Thus, choosing a fluorescent variant with a lower tendency to undergo oligomerization (e.g., mCherry, mCherry2, mCitrine, and mScarlett) is recommended. Spectral characteristics and brightness are essential, especially when replisomes are localized together with other cellular components (e.g., chromosome and membrane) (Shaner et al., 2005). Importantly, FP are sensitive to pH and cannot be utilized to analyze anaerobic bacteria, as maturation of the chromophore requires oxygen molecules (Shaner et al., 2005; Landete et al., 2015). Fluorescent fusion proteins are suitable for both qualitative long-term live cell imaging and quantitative analysis. For example, Y-Pet fusion with a variety of replisome subunits was used to quantify the numbers of copies of particular proteins within a replication eye *in vivo* (Reyes-Lamothe et al., 2010). However, most of these variants lacked the properties required for super-resolution imaging. In the latter case, proteins of interest should be fused with photoactivated or photoconvertible proteins. Recently published studies may provide hints regarding single-molecule resolution microscopy of replication complexes (Georgescu et al., 2012; Stracy et al., 2014; Liao et al., 2016; Lewis et al., 2017). The fusion of replisome subunits with HaloTag may be an alternative to FP. The size of HaloTag is similar to that of FP, but the ligands that bind to HaloTag have better fluorescence yield, resulting in a higher signal compared with standard FPs (HaloTag^®^ Protein Purification System, 2018). The advantage of using direct fluorescent ligands (e.g., dTMR and dR110) is that they do not need to be washed out before acquisition. Halo ligands are also suitable for high-resolution microscopy.

Replication tracking (particularly initiation of replication) is often accompanied by localization of nascent *oriCs* (Figure 2B). The fluorescence repressor operator system (FROS) or ParB/*parS* is frequently used for live cell tracking (Lau et al., 2003). The FROS system (Figure 2B) consists of two components: operator sequences (usually *lacO* or *tetO* arrays repeated up to several hundred times in tandem and interspersed by oligonucleotide spacers) and an FP-tagged repressor protein (LacI-FP or TetR-FP), which binds to the operator sequences. FROS was efficiently used to localize chromosomal loci, including *oriC*, terminus and other specific loci on both replichores in a variety of species (Viollier et al., 2004; Fogel and Waldor, 2005; Frunzke et al., 2008; Liu et al., 2010; Vallet-Gely and Boccard, 2013; Wang et al., 2014; Santi and McKinney, 2015). However, it is often difficult to insert the large operator arrays into the chromosome, particularly in highly transcribed regions such as *oriC* (Le and Laub, 2014). Moreover, overexpression of repressor may result in replication/transcription hold-up or alteration in segregation of replicated regions (Possoz et al., 2006; Mettrick and Grainge, 2016). Thus, low levels of repressor should be produced, usually by using inducible promoters. Additionally, tracking *oriCs* together with replisomes requires delivery of the repressor-FP fusion protein from the chromosomal locus, either as a part of an operator array construct or inserted into an attachment site. Although FROS may provide invaluable data, its instability is a major drawback.

The ParB-FP/*parS* system (which originated from naturally existing chromosome and/or plasmid partitioning strategies) (Figure 2B) represents an easier alternative to FROS. This system uses an intrinsic feature of ParB, its binding to centromere-like *parS* sequences (Wang et al., 2011; Reyes-Lamothe et al., 2012; Badrinarayanan et al., 2015). Most bacterial species possess the ParAB*S* chromosome segregation system, except for several well-studied Gammaproteobacteria, including *E. coli*. Because most chromosomal *parS* sites are localized proximal to the *oriC*-proximal regions (Livny et al., 2007), introduction of fluorescent ParB, which oligomerizes within *parS* sequences, addresses all of the system requirements for successful *oriC* labeling. This approach has been shown effective in a number of bacteria, including *Mycobacterium*, *M. xanthus* (Harms et al., 2013), *Streptomyces* (Donczew et al., 2016; Kois-Ostrowska et al., 2016), *C. crescentus* (Laloux and Jacobs-Wagner, 2013), and *C. glutamicum* (Donovan et al., 2010; Böhm et al., 2017). In bacteria lacking a chromosomal ParAB*S* system (e.g., *E. coli*), plasmid-derived partitioning components (phage P1 or *Yersinia pestis* MT1ParB/*parS* systems) are frequently used (Youngren et al., 2000; Li et al., 2002; Nielsen et al., 2006, 2007). The use of plasmid-derived *parS*/ParB is also beneficial, as it does not interfere with the endogenous chromosomal ParAB*S* system or another plasmid-derived *parS*/ParB system (P1/MT1), allowing the simultaneous localization of multiple chromosomal loci. Its major advantage compared with FROS is that insertion of only a few copies of *parS* is sufficient for strong fluorescent signals after ParB-FP binding.

Determination of the specific point (and subcellular localization) at which replication is initiated requires long-term imaging of living cells (from several minutes to hours, depending on the bacterial growth rate and the conditions being tested, e.g., rich versus minimal medium). The simplest way to analyze replication at the single-cell level is to spread the cells of the reporter strain on the agar pad (a thin agar layer between the microscope slide and the cover glass) or on the bottom of solidified medium inside culture dishes (Joyce et al., 2011; Dhar and Manina, 2015). Although simple and low-cost, this approach is not always applicable (e.g., labeling and medium changing). Microfluidic flow chambers are used for the latter purposes, as well as for rapidly changing culture conditions (e.g., applying stress). Various microfluidic chips and plates are commercially available from an increasing number of companies, whereas custom made (usually PDMS) chips are a cost-reducing alternative and also allow for more personalized applications (Wang et al., 2010; Cattoni et al., 2013; Dhar and Manina, 2015; Trojanowski et al., 2015; Wallden et al., 2016). The architecture of microfluidic chips and plates varies among studies and choosing the right one should be dictated by the specific study purpose and the availability of additional equipment, e.g., peristaltic/syringe/pressure pumps, flow controllers, or automation.

## Spatiotemporal Localization of the Replisome During Replication Initiation

**FIGURE 3:**
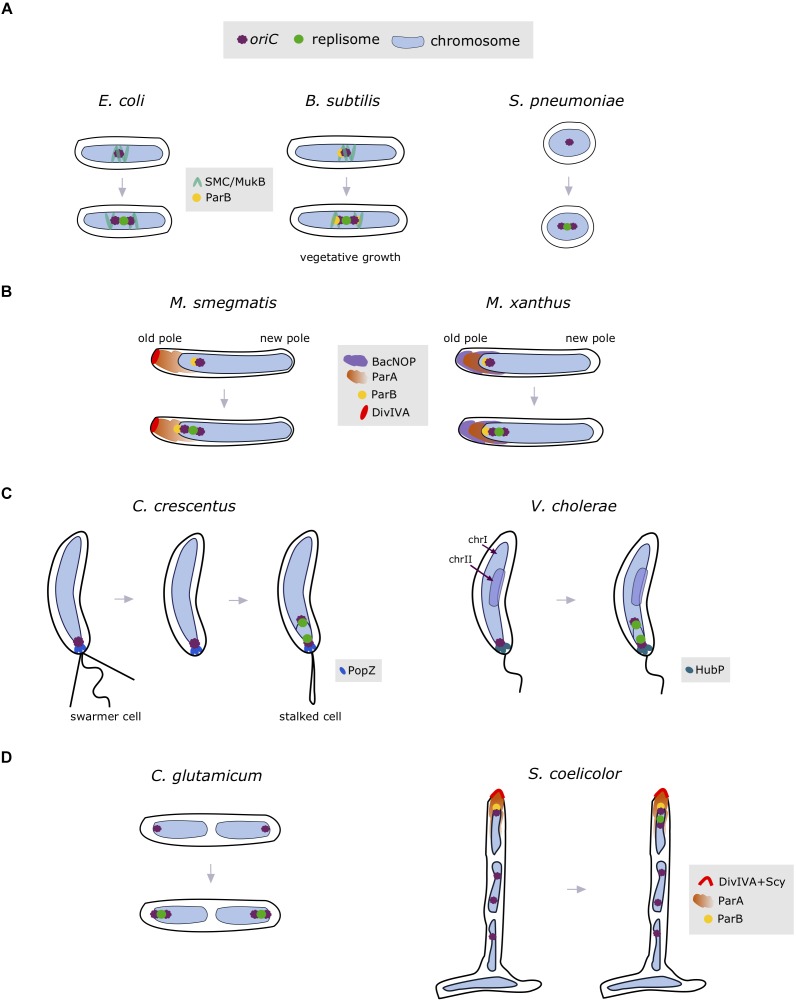
Spatial organization of the chromosome entails positioning of the site of replisome assembly. Bacteria exhibiting a *left-ori-right* orientation start replication in mid-cell **(A)**, where the *oriC* region is organized by the condensins SMC/MukB (marked in green) and ParB (indicated as yellow circle). Off-centered replisome positioning **(B)** is associated with complex interactions between *oriC* and the ParAB*S* system (*M. smegmatis*; ParA indicated as orange cloud, ParB in yellow circle and DivIVA in red) or bactofilins (*M. xanthus*; BacNOP depicted as violet cloud). In the *ori-ter* organized chromosomes, replication is initiated at the cell pole **(C)**, at which the *oriC* region is anchored by specific proteins (i.e., PopZ in *C. crescentus* and HubP in *V. cholerae* indicated as blue and marine blue ovals, respectively). **(D)** Subpolar positioning of replisomes has also been observed in the multiploid bacteria *S. coelicolor* (ParA indicated as orange cloud and polarisome complex proteins: ParB and DivIVA interacting with Scy depicted in yellow and red, respectively) and *C. glutamicum*. *OriC* region(s) and replisome(s) are indicated as violet and green circles, while chromosome is depicted in light blue.

Localization of the replication machinery at the beginning of DNA synthesis is dependent on *oriC* position, and is therefore connected with the spatial arrangement of the chromosome. In bacteria having *oriC* and *ter* regions positioned at the mid-cell, the intervening chromosomal regions (i.e., the left and right chromosomal arms) are stretched out toward opposite cell poles, creating a *left-ori-right* pattern, whereas cells having *oriC* and *ter* regions localized to opposite poles show an *ori-ter* chromosomal arrangement (Wang and Rudner, 2014). Replisomes in the cells exhibiting a *left-ori-right* configuration are assembled in the mid-cell region of the chromosome. This pattern has been observed in *E. coli* cells (Postow et al., 2004; Valens et al., 2004; Boccard et al., 2005) and during the vegetative growth of *B. subtilis* (the chromosome in *B. subtilis* is oscillating between *left-ori-right* and *ori-ter* configuration) (Wang et al., 2014; Figure 3A). During sporulation, however, the *B. subtilis* chromosome adopts an *ori-ter* orientation to segregate an entire copy of the chromosome within each spore. Positioning of the *oriC* at the mid-cell of *B. subtilis* and *E. coli* is maintained by the condensins SMC and MukB (a structural homolog of SMC), respectively (Niki et al., 1992; Danilova et al., 2007; Sullivan et al., 2009). SMC can compact large chromosomal regions, and, by interacting with ParB protein, organizes the *oriC*-proximal regions in *B. subtilis*, with ParB binding to *parS* sequences located near *oriC* (Gruber and Errington, 2009). The interaction of MukB with the nucleoid associated protein HU ensures proper *oriC* positioning in *E. coli* cells (Lioy et al., 2018). After initiation, *E. coli* replisomes oscillate near the cell center, while newly replicated *oriC*s are segregated toward the cell poles (Reyes-Lamothe et al., 2008). In comparison, *B. subtilis* replisomes colocalize throughout replication (Migocki et al., 2004), and are therefore visible as a single fluorescent focus. Replisome positioning in the cell center can be also found in oval-shaped *S. pneumoniae* (Kjos and Veening, 2014; van Raaphorst et al., 2017), which, similar to many other bacteria including *B. subtilis*, encodes an SMC homolog.

Some bacteria, such as *M. smegmatis* (Santi and McKinney, 2015; Trojanowski et al., 2015) and *M. xanthus* (Harms et al., 2013), exhibit off-center replisome localization during the initiation of replication (see Figure 3B). In *M. smegmatis*, segregation of the newly replicated *oriC*s starts immediately after initiation of replication, with one *oriC* remaining near the old cell pole and the other traveling toward the opposite pole (Ginda et al., 2017; Hołówka et al., 2018). Replisomes oscillate in the old-pole-proximal cell half during most of the replication process, but localize closer to the new cell pole prior to termination (Trojanowski et al., 2015). A slight asymmetry in mycobacterial replisome positioning is associated with the apical growth mode of these bacteria. Positioning of *oriC* region(s) in *Mycobacterium* depends on the interaction of ParB with ParA protein, which in turn interacts with the polar growth determinant, DivIVA protein (Ginda et al., 2013).

As a result of the asymmetric location of *oriC*, *M. xanthus* replisomes are positioned at the subpolar regions (Figure 3B; Harms et al., 2013). Although *M. xanthus* contains a DivIVA homolog, suggesting analogous interactions at the pole as described for *Mycobacterium*, deletion of this homolog does not affect cell division or chromosome segregation. Rather, localization of the ParA and ParB-*parS* complexes (and thus the *oriC* region) in *M. xanthus* is controlled by the bactofilins BacNOP, through the direct interactions of ParA and ParB with the scaffold created by BacNOP (Lin et al., 2017).

Bacteria exhibiting complex life cycles often show an *ori-ter* chromosome orientation (Figure 3C). In *C. crescentus* stalked cells, chromosome replication starts at the old cell pole (Jensen et al., 2001). The anchorage of the chromosome at the old cell pole is maintained by the protein PopZ (Bowman et al., 2008). Similarly, in *V. cholerae*, the origin (*ori*I) of one of the two chromosomes, chrI, is attached to the old pole by HubP protein (Yamaichi et al., 2012), thereby setting the subcellular position for assembly of the replication machinery. In contrast, the origin (*ori*II) of the second, smaller chromosome (chrII) is located at mid-cell. Replication of *V. cholerae* chrII starts later than that of chrl to synchronize the termination of replication of both chromosomes (Demarre et al., 2014; Ramachandran et al., 2018). As a result of the subpolar localization of *C. crescentus* and *V. cholerae* (chrI) replisomes near the old cell pole, one of the newly replicated *oriC* regions travels across the chromosome to the opposite cell pole with the assistance of the ParAB*S* system (Toro et al., 2008; Ramachandran et al., 2014). Interestingly, in *P. aeruginosa* exhibiting *ori-ter* orientation, the chromosome is apparently not anchored to the cell pole, as shown by the cytoplasmic gap between *oriC* and the cell pole (Vallet-Gely and Boccard, 2013).

The multiploid and apically growing bacterial species *S. coelicolor*, exhibits another mode of spatiotemporal replisome localization, in which replication is initiated during vegetative growth (Figure 3D; Kois-Ostrowska et al., 2016). Replication of multiple copies of the *S. coelicolor* chromosome starts asynchronously, and newly replicated sister chromosomes follow the extending hyphal tip. Similar to *Mycobacterium*, positioning of the tip-proximal *oriC* (and hence the replisomes) is maintained through ParA interactions with the polarisome complex, which includes the proteins ParB, DivIVA, and Scy (Flärdh et al., 2012; Ditkowski et al., 2013). In the closely related and diploid species *C. glutamicum*, replisomes are assembled on each chromosome asymmetrically, in proximity to the cell poles (Figure 3D; Böhm et al., 2017). Fluorescently tagged ParB attaches to the cell poles, suggesting an *ori-ter-ter-ori* spatial orientation of *C. glutamicum* chromosomes.

Described differences among bacteria in the positioning of *oriC* regions during the replication initiation reflect the different modes of chromosome segregation. Mid-cell replisomes location results in symmetric segregation of *oriC*s toward the opposite cell poles, while polar and off-center replisome positioning imply asymmetric segregation of the newly replicated *oriC* regions. Furthermore, polar localization requires the complex system to either anchor *oriC* directly at the pole (e.g., PopZ and HubP proteins) or to maintain the subpolar position by protein complexes (e.g., the interaction of ParAB*S* system with the DivIVA or the BacNOP). Such variety in the composition of multiprotein complexes involved in *oriC*(s) positioning provides an opportunity for the discovery of novel genus/species-specific drug targets.

## Conclusion

Single-cell fluorescence imaging and fluorescence tagging techniques allow researchers to precisely visualize proteins and their complexes inside living bacterial cells in real time. These techniques revealed that many proteins are targeted to distinct subcellular positions, where they participate in various cellular processes including chromosome replication. Recent studies using advanced live-cell imaging demonstrated that chromosome replication is coordinated with other key steps of the cell cycle, such as chromosome segregation and cell division. Proteins (or protein complexes) involved in condensation (i.e., SMC/MukB), chromosome segregation (i.e., ParAB in Gram-negative and Gram-positive bacteria) and/or cell division (DivIVA in Gram-positive bacteria) take part directly or indirectly in *oriC* positioning, thus indicating the site of replisome assembly. Additionally, other proteins guiding the *oriC* region have been recently identified. Interestingly, they vary significantly among different bacteria, e.g., PopZ (*C. crescents*), HubP (*V. cholerae*, chromosome I), and bactofilins (*M. xanthus*). The diversity and complexity of the systems involved in *oriC* (and thus replisome) subcellular positioning suggest the possibility of developing new antimicrobial therapies and/or altering existing treatments (Kaguni, 2018).

## Author Contributions

All authors listed have made a substantial, direct, and intellectual contribution to the work, and approved it for publication.

## Conflict of Interest Statement

The authors declare that the research was conducted in the absence of any commercial or financial relationships that could be construed as a potential conflict of interest.
